# Supplementary material for: Hamiltonian of a flux qubit-LC oscillator circuit in the deep–strong-coupling regime
Source: Sci Rep. 2022 Apr 26;12:6764. doi: 10.1038/s41598-022-10203-1 (PMC9042887; doi:10.1038/s41598-022-10203-1)
Supplement: Supplementary file 1 — Supplementary Information. [file 41598_2022_10203_MOESM1_ESM.pdf]

Supplementary Information: Hamiltonian of a flux qubit-LC oscillator circuit in the deep-strong-coupling regime

F. Yoshihara,[1,*] S. Ashhab,[1,2] T. Fuse,[1] M. Bamba,[3,4] and K. Semba[1,†]

[1] *Advanced ICT Research Institute, National Institute of Information and Communications Technology, 4-2-1, Nukuikitamachi, Koganei, Tokyo 184-8795, Japan*

[2] *Qatar Environment and Energy Research Institute, Hamad Bin Khalifa University, Qatar Foundation, Doha, Qatar*

[3] *Department of Physics, Kyoto University, Kyoto 606-8502, Japan*

[4] *PRESTO, Japan Science and Technology Agency, Kawaguchi 332-0012, Japan*

(Dated: April 12, 2022)

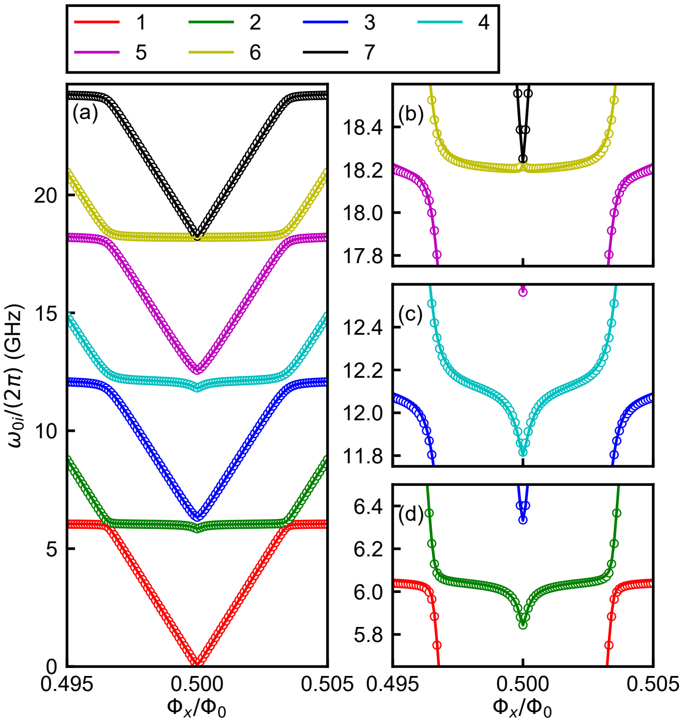

FIG. S1. (a) Transition frequencies of the qubit-oscillator circuit up to the seventh excited state for $L_c = 350$ pH. Numerically calculated transition frequencies from $\hat{\mathcal{H}}_{circ}$ are plotted as circles, while the results of the fitting by $\hat{\mathcal{H}}_R$ are plotted as lines. The parameters of $\hat{\mathcal{H}}_R$ obtained by fitting the spectra of $\hat{\mathcal{H}}_{circ}$ to $\hat{\mathcal{H}}_R$ are $\omega/(2\pi) = 6.054$ GHz, $\Delta_q/(2\pi) = 2.133$ GHz, $g/(2\pi) = 7.562$ GHz, and $I_p = 282.2$ nA. Red, green, blue, cyan, magenta, yellow, and black colors indicate transition frequencies ω_{01}, ω_{02}, ω_{03}, ω_{04}, ω_{05}, ω_{06}, and ω_{07}, respectively. (b), (c), (d) The same spectra with smaller range of frequency around 3ω, 2ω, and ω.

S1. FITTING OF $\mathcal{H}_{circ}$ UP TO THE SEVENTH EXCITED STATE

Transition frequencies of the qubit-oscillator circuit numerically calculated from $\hat{\mathcal{H}}_{circ}$ up to the seventh excited state are plotted in Figure S1 for $L_c = 350$ pH using the same circuit parameters as in Fig. 3. The calculated spectra can still be fitted well by $\hat{\mathcal{H}}_R$. In the fitting, $\hat{\mathcal{H}}_R$ are numerically diagonalized and then the parameters ω, Δ_q, g, and I_p, are varied to obtain the best fit. The average of the squares of the residuals in the least-squares method of obtaining the parameters of $\hat{\mathcal{H}}_R$ by fitting, $\overline{[\delta\omega_{0i}/(2\pi)]^2}$ (i = 1,2,3,4,5,6, and 7), is 152 MHz^2.

* fumiki@nict.go.jp

† Present address: Institute for Photon Science and Technology, The University of Tokyo, Tokyo 113-0033, Japan.

This value is almost 10 times larger than the case of the fitting up to the third excited state, but still within a moderate level considering that the largest transition frequency is more than 20 GHz.

S2. GAUGE TRANSFORMATION

The circuit Hamiltonian in the flux gauge $\hat{\mathcal{H}}_{circ}$ can be written as

$$\begin{aligned}\hat{\mathcal{H}}_{circ} &= \frac{\hat{q}_1^2}{2C} + \frac{\hat{q}_2^2}{2C_J} - E_J \cos\left(2\pi\frac{\hat{\Phi}_2 - \Phi_x}{\Phi_0}\right) + \frac{\hat{\Phi}_1^2}{2L_{LC}} + \frac{\hat{\Phi}_2^2}{2L_{FQ}} - \frac{\hat{\Phi}_1\hat{\Phi}_2}{L_{12}} \\ &= \frac{\hat{q}_1^2}{2C} + \frac{\hat{q}_2^2}{2C_J} - E_J \cos\left(2\pi\frac{\hat{\Phi}_2 - \Phi_x}{\Phi_0}\right) \\ &\quad + \frac{1}{2L_{LC}}\left(\hat{\Phi}_1 - \frac{L_{LC}}{L_{12}}\hat{\Phi}_2\right)^2 + \frac{1}{2}\left(\frac{1}{L_{FQ}} - \frac{L_{LC}}{L_{12}^2}\right)\hat{\Phi}_2^2. \end{aligned} \tag{S1}$$

The Hamiltonian can now be transformed into the charge gauge rather easily. First we note that the unitary operator

$$\hat{\mathcal{U}} = \exp\left(\frac{1}{i\hbar}\alpha\hat{\Phi}_2\hat{q}_1\right) \tag{S2}$$

transforms the flux and charge operators as follows:

$$\hat{\mathcal{U}}^\dagger\hat{\Phi}_1\hat{\mathcal{U}} = \hat{\Phi}_1 + \alpha\hat{\Phi}_2, \tag{S3}$$

$$\hat{\mathcal{U}}^\dagger\hat{\Phi}_2\hat{\mathcal{U}} = \hat{\Phi}_2, \tag{S4}$$

$$\hat{\mathcal{U}}^\dagger\hat{q}_1\hat{\mathcal{U}} = \hat{q}_1, \tag{S5}$$

$$\hat{\mathcal{U}}^\dagger\hat{q}_2\hat{\mathcal{U}} = \hat{q}_2 - \alpha\hat{q}_1. \tag{S6}$$

Then, if we set $\alpha = L_{LC}/L_{12}$, the circuit Hamiltonian is transformed into

$$\begin{aligned}\hat{\mathcal{H}}'_{circ} &= \hat{\mathcal{U}}^\dagger\hat{\mathcal{H}}_{circ}\hat{\mathcal{U}} \\ &= \frac{\hat{q}_1^2}{2C} + \frac{1}{2C_J}\left(\hat{q}_2 - \frac{L_{LC}}{L_{12}}\hat{q}_1\right)^2 \\ &\quad - E_J\cos\left(2\pi\frac{\hat{\Phi}_2 - \Phi_x}{\Phi_0}\right) + \frac{\hat{\Phi}_1^2}{2L_{LC}} + \frac{1}{2}\left(\frac{1}{L_{FQ}} - \frac{L_{LC}}{L_{12}^2}\right)\hat{\Phi}_2^2 \\ &= \left(\frac{1}{2C} + \frac{L_{LC}^2}{2C_J L_{12}^2}\right)\hat{q}_1^2 + \frac{\hat{\Phi}_1^2}{2L_{LC}} + \frac{1}{2C_J}\hat{q}_2^2 \\ &\quad + \frac{1}{2}\left(\frac{1}{L_{FQ}} - \frac{L_{LC}}{L_{12}^2}\right)\hat{\Phi}_2^2 - E_J\cos\left(2\pi\frac{\hat{\Phi}_2 - \Phi_x}{\Phi_0}\right) - \frac{L_{LC}}{C_J L_{12}}\hat{q}_1\hat{q}_2. \end{aligned} \tag{S7}$$

S3. ENERGY SHIFTS UP TO SECOND ORDER IN PERTURBATION THEORY

To investigate the reason for the poor reproducibility of the characteristic spectra of $\hat{\mathcal{H}}'_{circ}$ by $\hat{\mathcal{H}}'_{R}$, we calculate the energy shifts of the four lowest energy levels using perturbation theory. Let us consider the Hamiltonian $\hat{\mathcal{H}}_{circ,\lambda} \equiv \hat{\mathcal{H}}_1 + \hat{\mathcal{H}}_2 + \lambda\hat{\mathcal{H}}_{12}$. Here, we assume that the eigenenergies and the eigenstates of $\hat{\mathcal{H}}_{circ,\lambda}$ can be described by a power series in λ as $E_{ni,\lambda} = \sum_{k=0}^{\infty} \lambda^k E_{ni}^{(k)}$ and $|ni\rangle_\lambda = \sum_{k=0}^{\infty} \lambda^k \left|ni^{(k)}\right\rangle$, where $E_{ni}^{(0)}$ and $\left|ni^{(0)}\right\rangle$ are the eigenenergies and the eigenstates of the non-interacting Hamiltonian $\hat{\mathcal{H}}_1 + \hat{\mathcal{H}}_2$, n is the number of photons in the oscillator, and $i = g, e$ represents the eigenstate of $\hat{\mathcal{H}}_2$. By taking the eigenenergies and the eigenstates of $\hat{\mathcal{H}}_{circ,\lambda}$ and multiplying by $\left\langle mj^{(k)}\right|$ from the left, the correction terms can be written as

$$E_{ni}^{(1)} = \left\langle ni^{(0)}\right| \hat{\mathcal{H}}_{12} \left|ni^{(0)}\right\rangle \tag{S8}$$

and

$$E_{ni}^{(2)} = \sum_{m,j} \frac{\left|\left\langle mj^{(0)}\middle|\hat{\mathcal{H}}_{12}\middle| ni^{(0)}\right\rangle\right|^2}{E_{ni}^{(0)} - E_{mj}^{(0)}} \equiv \sum_{m,j} \hbar\chi_{ni,mj}. \tag{S9}$$

Here, $\chi_{ni,mj}$ is the energy shift of the state $|ni\rangle$ due to the interaction with the state $|mj\rangle$, where $m = 0, 1, 2, \dots,$ $j = g, e, f, h, \dots,$ and $|f\rangle$ and $|h\rangle$ respectively represent the second and third excited states of $\hat{\mathcal{H}}_2$. We find that $E_{ni}^{(1)} = 0$ for all the combinations of $n = 0, 1$ and $i = g, e$, and the total energy shifts up to the second-order perturbation χ_{ni} are simply determined by the second-order correction terms: $\chi_{ni} = \sum_{m,j} \chi_{ni,mj}$. We also find that the third- or higher-order correction terms are much smaller than the second-order correction term: the numerator of the nth order correction term is the product of n matrix elements of $\hat{H}_{12}$, which are at most several hundred MHz, while the denominator of the nth order correction term is the product of $n-1$ energy-level differences, which are in the order of GHz or more.

Figures S2(a)-(d) show nonzero energy level shifts $\chi_{ni,mj}$ of the non-interacting Hamiltonian $\hat{\mathcal{H}}_1 + \hat{\mathcal{H}}_2$ due to the interaction term $\hat{\mathcal{H}}_{12}$ for the lowest four eigenstates $|ni\rangle = |0g\rangle$, $|0e\rangle$, $|1g\rangle$, and $|1e\rangle$ of the non-interacting Hamiltonian $\hat{\mathcal{H}}_1 + \hat{\mathcal{H}}_2$ for $L_c = 20$ pH. Although some energy shifts $\chi_{ni,mj}$ appear to be equal to zero everywhere, they are in fact finite but extremely small. The level shifts $\chi_{ni,mj}$ that involve the higher qubit levels $j = f, h$ plotted

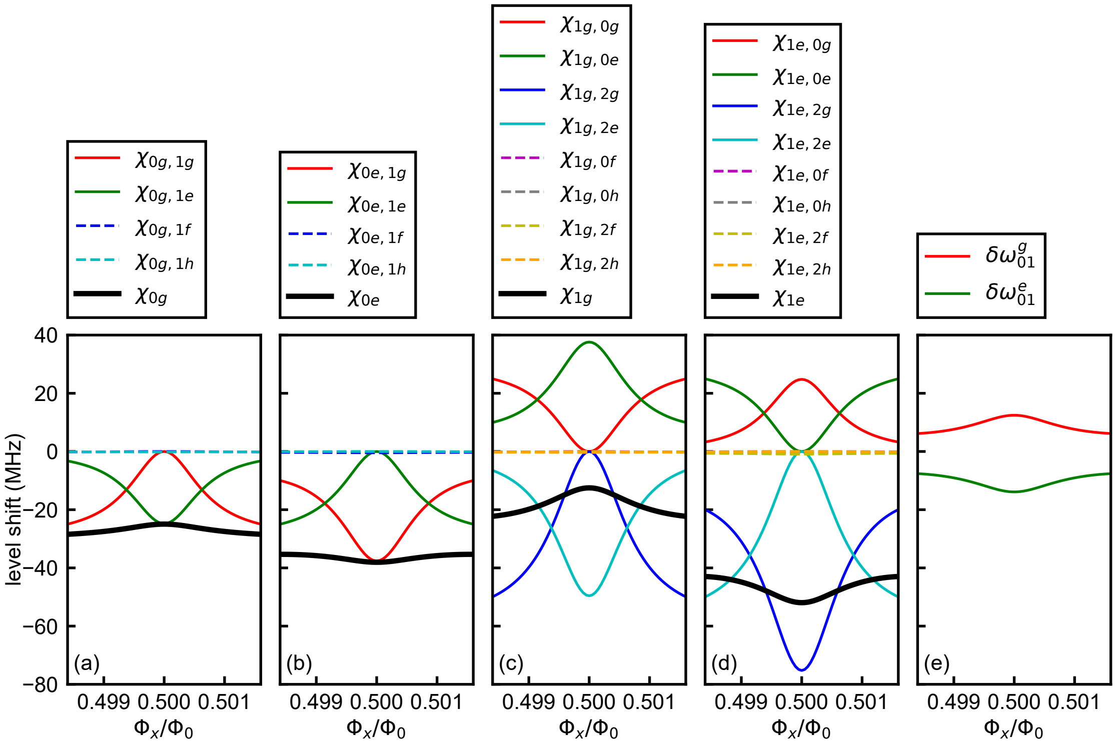

FIG. S2. Nonzero energy level shifts $\chi_{ni,mj}$ of the non-interacting Hamiltonian $\hat{\mathcal{H}}_1 + \hat{\mathcal{H}}_2$ due to the term $\hat{\mathcal{H}}_{12}$ and the state $|mj\rangle$ for the four lowest eigenstates $|ni\rangle$ = (a) $|0g\rangle$, (b) $|0e\rangle$, (c) $|1g\rangle$, and (d) $|1e\rangle$ for $L_c = 20$ pH. (e) the net change of the transition frequencies $\delta\omega_{01}^{g} = \chi_{1g} - \chi_{0g}$ and $\delta\omega_{01}^{e} = \chi_{1e} - \chi_{0e}$, obtained by combining the shifts in Panels (a)-(d).

using dashed lines are all close to zero, and the net level shifts are almost completely determined by the eigenstates involving the two lowest energy levels of $\hat{\mathcal{H}}_2$. Figure S2(e) shows the net change of the transition frequencies $\delta\omega_{01}^{g} = \chi_{1g} - \chi_{0g}$ and $\delta\omega_{01}^{e} = \chi_{1e} - \chi_{0e}$ from the resonance frequency of the oscillator described by $\hat{\mathcal{H}}_1$, which reproduce the peaks and dips that occur in the spectrum at $\Phi_x/\Phi_0 = 0.5$ shown in Fig. 4**a** in the main text.

We now consider the case of the circuit Hamiltonian $\hat{\mathcal{H}}'_{circ}$. Figures S3(a)-(d) show nonzero energy level shifts $\chi_{ni,mj}$ of the non-interacting Hamiltonian $\hat{\mathcal{H}}'_1 + \hat{\mathcal{H}}'_2$ due to the interaction with the state $|mj\rangle$ for eigenstates $|ni\rangle$ ($n = 0, 1,\ i = g, e$) for $L_c = 20$ pH. Figure S3(e) shows the net change of the transition frequencies $\delta\omega_{01}^{g} = \chi_{1g} - \chi_{0g}$ and $\delta\omega_{01}^{e} = \chi_{1e} - \chi_{0e}$ from the resonance frequency of the oscillator described by $\hat{\mathcal{H}}'_1$. Although the qualitative agreement is still not excellent, the peaks and dips that occur in the spectrum at Φ_x/Φ_0 are qualitatively reproduced. The agreement of $\delta\omega_{01}^{g}$ is better than that of $\delta\omega_{01}^{e}$. Contrary to

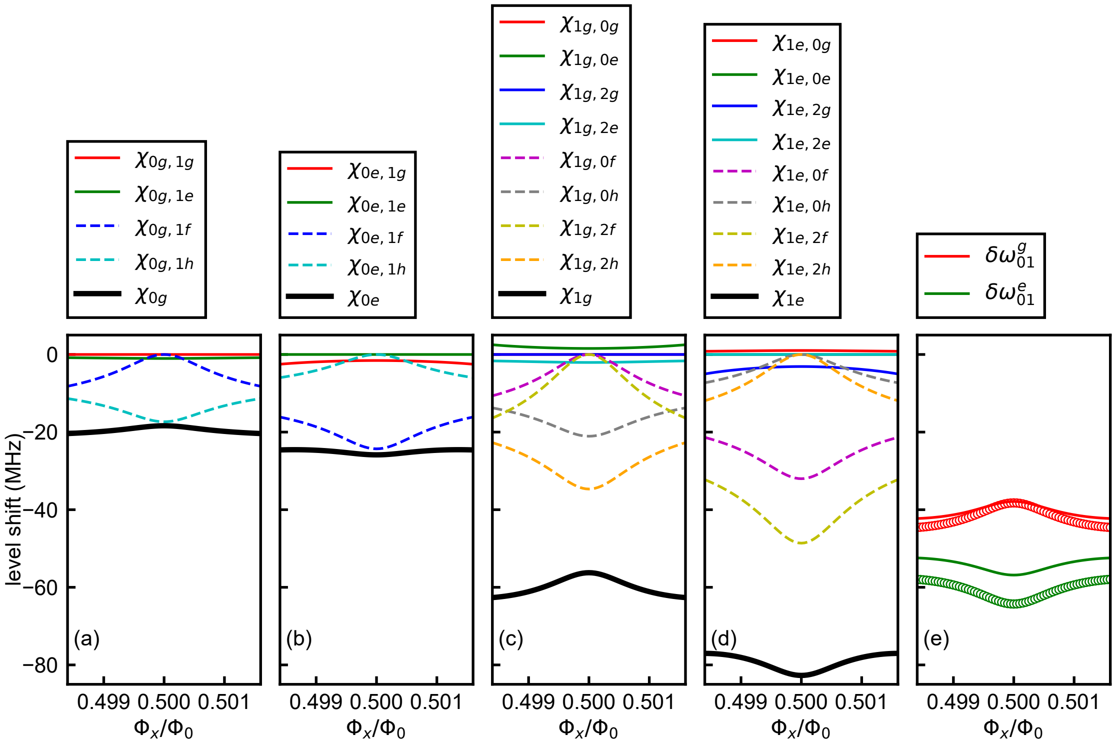

FIG. S3. Nonzero energy level shifts $\chi_{ni,mj}$ of the non-interacting Hamiltonian $\hat{\mathcal{H}}_1' + \hat{\mathcal{H}}_2'$ due to the term $\hat{\mathcal{H}}_{12}'$ and the state $|mj\rangle$ for the four lowest eigenstates $|ni\rangle =$ (a) $|0g\rangle$, (b) $|0e\rangle$, (c) $|1g\rangle$, and (d) $|1e\rangle$ for $L_c = 20$ pH. (e) the net changes of the transition frequencies $\delta\omega_{01}^{g} = \chi_{1g} - \chi_{0g}$ and $\delta\omega_{01}^{e} = \chi_{1e} - \chi_{0e}$, obtained by combining the shifts in Panels (a)-(d). Numerically calculated transition frequencies of $\hat{\mathcal{H}}_{circ}$, $\omega_{02} - \omega'$ and $\omega_{13} - \omega'$, are plotted as circles, where ω' is the resonance frequency of $\hat{\mathcal{H}}_{circ}'$.

the case of the circuit Hamiltonian $\hat{\mathcal{H}}_{circ}$, the level shifts $\chi_{ni,mj}$ that involve the higher qubit levels $j = f, h$ plotted using dashed lines are larger than those including $j = g, e$ in most of the flux bias conditions, and the level shifts are mainly determined by the third and the fourth lowest energy levels of $\hat{\mathcal{H}}_2'$. These results explain the difference between the spectra of $\hat{\mathcal{H}}_{circ}'$ and those of $\hat{\mathcal{H}}_R'$, in which $\hat{\mathcal{H}}_{FQ}'$ contains only the lowest two energy levels, as shown in Fig. 4**e** in the main text: The spectrum represented by solid grey and red lines obtained from $\hat{\mathcal{H}}_R'$ are similar to those of an uncoupled qubit-oscillator circuit, whereas the spectrum represented by the open grey and red circles obtained from $\hat{\mathcal{H}}_{circ}'$ show the peaks and dips around the symmetry point and the overall frequency is lower.

S4. MATRIX ELEMENTS OF THE CHARGE AND FLUX OPERATORS

In Section S3, the energy level shifts of the non-interacting Hamiltonian caused by the interaction term were studied using perturbation theory. The energy shifts of the states $|ni\rangle$ are given in Eq. (S9). Considering that the states $\left|ni^{(0)}\right\rangle$ and $\left|mj^{(0)}\right\rangle$ are separable, the following matrix elements can be written as products of matrix elements of the oscillator and the qubit:

$$\left\langle mj^{(0)} \left| \hat{\Phi}_1 \hat{\Phi}_2 \right| ni^{(0)} \right\rangle = \left\langle m \left| \hat{\Phi}_1 \right| n \right\rangle \left\langle j \left| \hat{\Phi}_2 \right| i \right\rangle \tag{S10}$$

and

$$\left\langle mj^{(0)} \right| \hat{q}_1 \hat{q}_2 \left| ni^{(0)} \right\rangle = \left\langle m \left| \hat{q}_1 \right| n \right\rangle \left\langle j \left| \hat{q}_2 \right| i \right\rangle . \tag{S11}$$

The matrix elements of the oscillator operators $\hat{\Phi}_1$ and $\hat{q}_1$ are analytically given as

$$\begin{aligned} \left\langle m \left| \hat{\Phi}_1 \right| n \right\rangle &= L_{LC} I_{zpf} \left\langle m \left| (\hat{a} + \hat{a}^\dagger) \right| n \right\rangle \\ &= L_{LC} I_{zpf} (\sqrt{n} \delta_{m,n-1} + \sqrt{n+1} \delta_{m,n+1}) \end{aligned} \tag{S12}$$

and

$$\begin{aligned} \left\langle m \left| \hat{q}_1 \right| n \right\rangle &= -iCV_{zpf} \left\langle m \left| (\hat{a} - \hat{a}^\dagger) \right| n \right\rangle \\ &= -iCV_{zpf} (\sqrt{n} \delta_{m,n-1} - \sqrt{n+1} \delta_{m,n+1}). \end{aligned} \tag{S13}$$

Only matrix elements that satisfy $m = n \pm 1$ are nonzero. The matrix elements of the qubit operators $\hat{\Phi}_2$ and $\hat{q}_2$ for $i = g, e$ and $j = g, e, f, h, k, l$, where $|k\rangle$ and $|l\rangle$ respectively represent the fourth and fifth excited states of $\hat{\mathcal{H}}_2$, are numerically calculated as shown in Fig. 6 in the main text. Note that the matrix elements of the qubit operators at $\varepsilon = 0$ and with a quadratic-plus-quartic potential energy function were studied in Ref. [1].

Regarding the matrix elements $\left| \left\langle j \left| \hat{\Phi}_2 \right| i \right\rangle \right|$ $(i = g, e)$, those involving the higher qubit levels $j = f, h, k, l$ are smaller than those of $j = g, e$. Together with the fact that the energy difference $\left| E_{ni}^{(0)} - E_{mj}^{(0)} \right|$ of $j = f, h, k, l$ is larger than those of $j = g, e$, which appears in the denominator of Eq. (S9), this result explains why the level shifts are almost completely determined by the eigenstates involving the two lowest energy levels of $\hat{\mathcal{H}}_2$. Regarding the matrix elements $|\langle j | \hat{q}_2 | i \rangle|$ $(i = g, e)$ on the other hand, some of those involving the higher qubit levels $j = f, h, k, l$ are significantly larger than those of $j = g, e$ in most of the flux

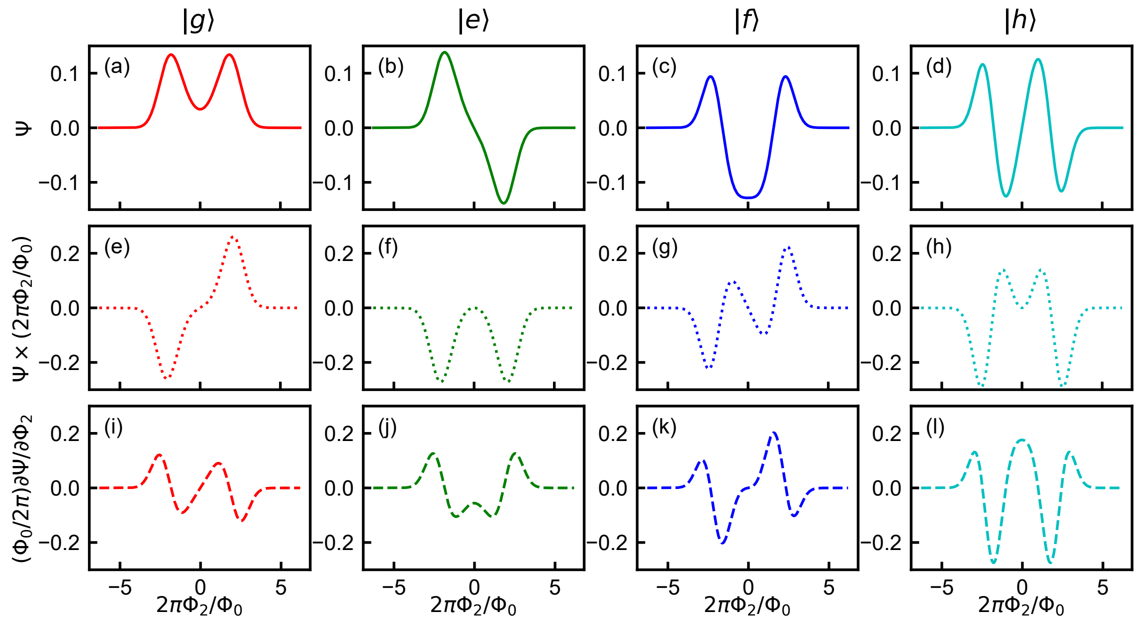

FIG. S4. Numerically calculated wavefunctions of the eigenstates of $\hat{\mathcal{H}}_2$, Ψ_i, as well as the combinations $\Psi_i \times (2\pi\Phi_2/\Phi_0)$, and $(\Phi_0/2\pi\Phi_2)\partial\Psi_i/\partial\Phi_2$ $(i = g, e, f, h)$, as functions of $2\pi\Phi_2/\Phi_0$ for $\Phi_x/\Phi_0 = 0.5$.

bias range. Although the energy difference $\left|E_{ni}^{(0)} - E_{mj}^{(0)}\right|$ is larger than those of $j = g, e$, the large matrix elements result in a situation where the level shifts are mainly determined by the third and fourth lowest energy levels of $\hat{\mathcal{H}}_2'$. We note here that the matrix elements involving the levels $j = k, l$ are smaller than those involving the levels $j = f, h$ and the energy difference is larger than those of $j = f, h$. Hence, the level shifts caused by higher levels with $j = k, l$ are smaller than those with $j = f, h$.

The relation between the different matrix elements can be intuitively understood using an analogy to a basic quantum physics problem. The qubit Hamiltonians $\hat{\mathcal{H}}_2$ and $\hat{\mathcal{H}}_2'$ have the same form as the Hamiltonian of a single particle in a trapping potential, with $\hat{\Phi}_2$ and $\hat{q}_2$ playing the roles of the position and momentum variables, respectively. Around the symmetry point, the trapping potential is a double-well potential. The energy eigenstates in the double-well potential are superpositions of the energy eigenstates in the two separate wells, which are approximately harmonic oscillator potentials. We therefore have superpositions of harmonic oscillator states whose centers are separated by a distance that is larger than their widths. Figures S4(a)-(d) show wave functions of the eigenstates of $\hat{\mathcal{H}}_2$, Ψ_i $(i = g, e, f, h)$, as functions of Φ_2 at the flux bias point $\Phi_x/\Phi_0 = 0.5$. Matrix elements of the position operator

can be written using the wave-function representation:

$$\left\langle j \left| \hat{\Phi}_2 \right| i \right\rangle = \int d\Phi_2 (\Psi_j^* \Phi_2 \Psi_i). \tag{S14}$$

Matrix elements that combine a pair of functions Ψ_j and $\Phi_2\Psi_i$ [Figs. S4(e)-(h)] with similar shapes are large. At the symmetry point, the matrix elements $\left|\left\langle e \left| \hat{\Phi}_2 \right| g \right\rangle\right| = \left|\left\langle g \left| \hat{\Phi}_2 \right| e \right\rangle\right| \sim \left|\left\langle h \left| \hat{\Phi}_2 \right| f \right\rangle\right| = \left|\left\langle f \left| \hat{\Phi}_2 \right| h \right\rangle\right|$ are large and the others are small or zero. Away from the symmetry point, the matrix elements $\left|\left\langle g \left| \hat{\Phi}_2 \right| g \right\rangle\right|$, $\left|\left\langle e \left| \hat{\Phi}_2 \right| e \right\rangle\right|$, $\left|\left\langle f \left| \hat{\Phi}_2 \right| f \right\rangle\right|$, and $\left|\left\langle h \left| \hat{\Phi}_2 \right| h \right\rangle\right|$ also become large. The matrix elements between states with $i = g, e$ and states with $j = f, h$ are therefore small compared to matrix elements involving only g and e. Similarly, the matrix elements of the momentum operator can be written using the wave-function representation:

$$\langle j \left| \hat{q}_2 \right| i \rangle = \int d\Phi_2 \left(\Psi_j^* \frac{\hbar}{i} \frac{\partial \Psi_i}{\partial \Phi_2} \right). \tag{S15}$$

Matrix elements that have a pair of functions Ψ_j and $\partial\Psi_i/\partial\Phi_2$ [Figs. S4(i)-(l)] with similar shapes are large. At the symmetry point, the matrix elements $|\langle h \left| \hat{q}_2 \right| g \rangle| = |\langle g \left| \hat{q}_2 \right| h \rangle| \sim |\langle f \left| \hat{q}_2 \right| e \rangle| = |\langle e \left| \hat{q}_2 \right| f \rangle|$ are large and the others are small or zero. Away from the symmetry point, the matrix elements $|\langle f \left| \hat{q}_2 \right| g \rangle|$, $|\langle g \left| \hat{q}_2 \right| f \rangle|$, $|\langle h \left| \hat{q}_2 \right| e \rangle|$, and $|\langle e \left| \hat{q}_2 \right| h \rangle|$ also become large. The matrix elements between states with $i = g, e$ and states with $j = f, h$ are therefore large compared to matrix elements involving only g and e.

[1] D. De Bernardis, P. Pilar, T. Jaako, S. De Liberato, and P. Rabl, Breakdown of gauge invariance in ultrastrong-coupling cavity QED, Phys. Rev. A **98**, 053819 (2018).
